# The vulnerary potential of botanical medicines in the treatment of bacterial pathologies in fish

**DOI:** 10.14202/vetworld.2021.551-557

**Published:** 2021-03-02

**Authors:** Farida Nurzhanova, Gaisa Absatirov, Bekzhasar Sidikhov, Alexander Sidorchuk, Nurbek Ginayatov, Kenzhebek Murzabaev

**Affiliations:** 1 Zhangir Khan West Kazakhstan Agrarian-Technical University, Republic of Kazakhstan; 2 Moscow State Academy of Veterinary Medicine and Biotechnology named after K.I. Skryabin, Russia

**Keywords:** fish, plant raw material, vulnerary effect, wound

## Abstract

**Background and Aim:**

The use of plant-based medicine in treating and preventing fish disease has become increasingly popular due to the resistance of bacterial pathogens to chemicals widely used in aquaculture. This study explored the vulnerary effect of botanical medicines made from local raw materials (greater celandine [*Chelidonium majus* L.], St. John’s wort [*Hypericum perforatum* L.], and bur beggar-ticks [*Bidens tripartita L*.]) in the treatment of sturgeon bacterial pathologies in a controlled environment.

**Materials and Methods:**

The vulnerary activity of herbal infusions was studied on spontaneously infected fish by assessing the degree of wound healing at regular intervals: The state of the wound, reduction of the wound surface area, the formation of granulation tissue, epithelization, and wound contraction.

**Results:**

A positive vulnerary effect of *C. majus*, *H. perforatum*, and *B. tripartita* was observed, consistent with the use of these plants in folk and traditional medicine. The plant materials eliminated infection, had anti-inflammatory and vulnerary effects, stimulated granulation tissue development, and enhanced regeneration. Compared with widely accepted methods (antibiotics and other chemotherapeutic agents), botanical medicine facilitated more effective treatment over the same period without side effects.

**Conclusion:**

Practical use and the results of this study show the potential of using herbal infusions for therapeutic purposes in aquaculture.

## Introduction

The aquaculture industry has experienced an emergence of diseases of various etiologies due to high stocking density and stressful conditions. Among the pathologies that occur when keeping fish in recirculating aquaculture systems, the most common are bacterial diseases caused by *Aeromonas* and *Pseudomonas* species [1-7].

Fish disease treatment strategies are based primarily on the use of antibiotics and chemotherapy. However, in recent years, plant-based medicines have become increasingly popular in aquaculture due to the resistance of bacterial pathogens to standard antibiotics, the accumulation of antibiotics in food fish, and environmental pollution.

Studies of the past decades showed encouraging results and opened up new opportunities for botanical therapies in fish [8-21]. Medicinal plants are used as immunostimulant, stress-relieving [10-12], antiparasitic [13,14], antioxidant, and antimicrobial agents [15-21].

Plant extracts of *Boesenbergia pandurata*, *Salmo ferox*, and *Zingiber zerumbet* for the prevention of illness caused by *Aeromonas hydrophila* and *Pseudomonas* spp. have a positive effect on the non-specific immune response and inhibit bacterial infection [18]. Bioactive compounds of *Origanum vulgare* have immunostimulant, cytotoxic, bactericidal, and antioxidant properties, which make them promising for fish aquaculture [19]. A study of *B. pandurata*, *S. ferox*, and *Z. zerumbet* showed their antibacterial properties in preventing and treating infections caused by *A. hydrophila* and *Pseudomonas fluorescens* [20]. Studies of the immunostimulant effects of brier (*Rosa canina*) on the hematological and immune parameters of sturgeons infected with *Mycobacterium salmoniphilum* showed a non-specific immune response and the antibacterial effect of this plant [21].

In addition to a wide range of anti-inflammatory, antimicrobial, and immunostimulant properties, biologically active plant components also exhibit vulnerary activity [22-29]. Plants and their extracts have great potential for the treatment of pathogenic and non-pathogenic wounds.

Data on the potential for wound healing of plant extracts used in aquaculture are scarce. There are studies on the wound healing effect of walnut leaves and onion leaves on wounds in *Clarias gariepinus* [30], accelerated wound healing in African catfish when *Ocimum gratissimum* was added to the feed [31], and wound healing in *Labeo rohita* fish with curcumin [32]. Healing in fish has mainly been studied in artificially modeled wounds or mechanical injuries. Little is known about the effect of plant extracts on the healing of wounds caused by pathogenic microorganisms.

This study explored the vulnerary effect of botanical medicines made from local raw materials (greater celandine [*Chelidonium majus* L.], St. John’s wort [*Hypericum perforatum* L.], and bur beggar-ticks [*Bidens tripartita* L.]) in the treatment of sturgeon bacterial pathologies in a controlled environment.

## Materials and Methods

### Ethical approval

The experimental plan and procedures with fish within the framework of the current research were approved by the local commission on biological ethics of TOO Batys Zoo Vet Servis (protocol No. 12 dated 01/10/2018). The experiments were conducted according to the recommendations set out in the “Rules of Good Clinical Practice of the Eurasian Economic Union” N 79, 03.11.2016.

### Study location and period

The study was conducted from September 2017 to January 2020 in the RAS at the “Educational and Scientific Complex of the Pilot Production of Aquaculture” LLP, where various species of sturgeon are grown in Uralsk, Republic of Kazakhstan.

### Plant material

The following medicinal plants were selected as raw materials for the botanical medicines used in the study: Greater celandine (*Chelidonium majus* L.) [24,28], St. John’s wort (*Hypericum perforatum* L.) [28,29], and bur beggar-ticks (*Bidens tripartita* L.) [27,28].

### Experimental conditions

The fish were kept in quarantine pools. During the experiment, the temperature in the closed water supply unit was maintained at an optimal level of 20-23°C. The main parameters of the aquatic environment corresponded to fishery standards: pH 7.0-8.0; dissolved oxygen 4.0 mg/L; permanganate oxidizability 10.0 mgO_2_/L; ammonia (NH4+) 0.5 mg/L; total hardness 6.0-8.0 mg/L; and biochemical oxygen demand 5 2.0 mgO_2_/L. When feeding the fish, an extruded compound feed was used, produced by the extrusion method, and consisting of fish meal (57.5%), soybean meal (20.0%), wheat grain (1.5%), fish oil (20.0%), and premix (1.0%). A One kg sample of compound feed contained 20.03 MJ of assimilated energy and 47.0% crude protein. Feeding was carried out by hand twice a day according to the calculated rates.

### Experimental models

To study wound healing, we used fish spontaneously (naturally) affected by pseudomonosis and aeromonosis, with moderate disease severity. Sturgeon individuals with pronounced clinical signs of a bacterial disease were identified during examination. Based on the clinical examination results, bacteriological studies were performed to confirm the preliminary diagnoses of pseudomonosis and aeromonosis.

Typical bacterial septicemia with ulceration and fin erosion, hemorrhagic ulcerative lesions on the skin (both small on the skin surface and penetrating deep into the muscles) were observed in sturgeons infected with pseudomonosis. Hemorrhagic septicemia was noted in fish infected with aeromonosis, characterized by abdominal distention and ascitic fluid accumulation, small superficial lesions, and local hemorrhage in the gills.

### Solution preparation

A stock solution was prepared from each plant material at a dilution of 1:20. Crushed plant material (50 g) was added to 1000 mL distilled water and infused in a boiling water bath for 30 min. The infusion was cooled, filtered, and used in therapeutic baths with an exposure of 30 min with a dilution of 500 mL of the initial solution per 50 L water.

### Experimental design

Three treatment groups were prepared: Group 1 (n=15, 53% with pseudomonosis, 47% with aeromonosis; *C. majus* infusion); Group 2 (n=15, 67% with pseudomonosis, 33% with aeromonosis; *B. tripartita* infusion); Group 3 (n=15, 60% with pseudomonosis, 40% with aeromonosis; *H. perforatum* infusion), and a control group (n=15, 53% with pseudomonosis, 47% with aeromonosis) were formed using the analog method. Fish in each experimental group were treated with herbal infusions every other day. The control group was not treated.

### Wound healing intensity

The healing process was observed by taking photographs, starting from the day the experiments began. The wound healing properties of the test plants were evaluated clinically (the state of the wound, reduction of the wound surface area (on average, mm^2^), the formation of granulation tissue, epithelization, and wound contraction) at regular intervals of 3, 5, 7, 10, and 15-20 days from the beginning of the experiment. Wounds were considered healed if the colliquative granulation tissue was no longer visible and the wound was covered with a new epithelium. Survival during the experiment was taken into account.

###  Statistical analysis

Digital data processing was carried out using the method of variation statistics using Microsoft Excel 2007.

## Results and Discussion

Clinical performance at the beginning of the experiment (day 0) in all experimental groups was as follows: Experimenters marked single or multiple ulcers of various surface areas and depths deep in the muscles with the formation of rims of bright red color along the edges on the dorsal, abdominal, and tail parts of the fish. Redness of the abdomen and the area around the anus was noted.

**Figure-1:**
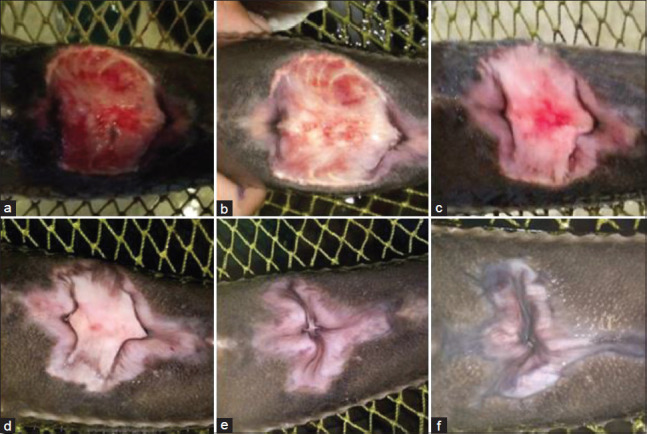
Macroscopic changes in the wound in the first experimental group (treatment with *Chelidonium majus*): (a) At the beginning of the experiments; (b) the 3^rd^ day of the experiments; (c) the 5^th^ day of the experiments; (d) the 7^th^ day of the experiments; (e) the 10^th^ day of the experiments; (f) the 15^th^ day of the experiments.

**Figure-2:**
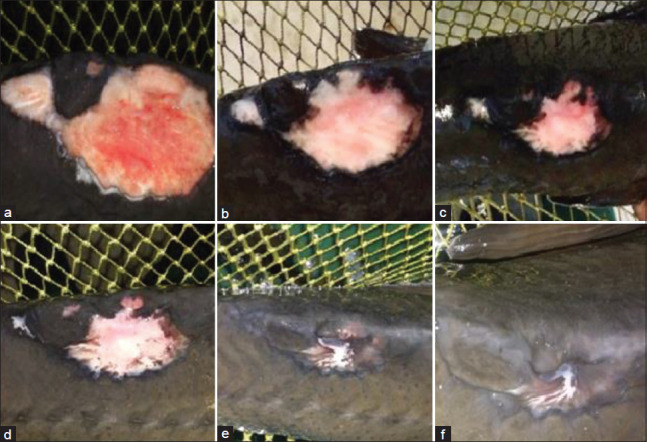
Macroscopic changes in the wound in the second experimental group (treatment with *Bidens tripartite*): (a) At the beginning of the experiments; (b) the 3^rd^ day of the experiments; (c) the 5^th^ day of the experiments; (d) the 7^th^ day of the experiments; (e) the 10^th^ day of the experiments; (f) the 15^th^ day of the experiments.

**Figure-3:**
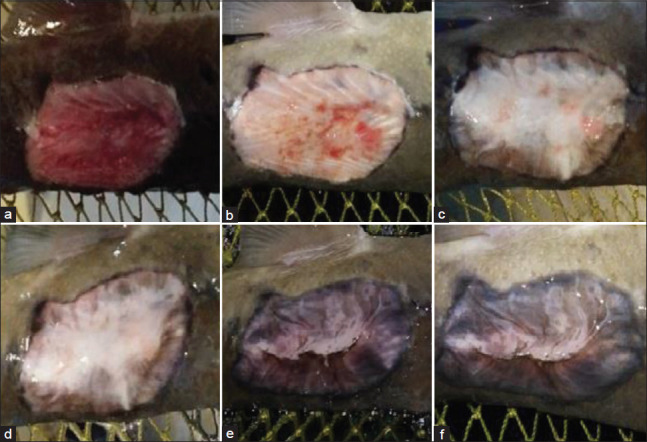
Macroscopic changes in the wound in the second experimental group (treatment with *Hypericum perforatum*): (a) At the beginning of the experiments; (b) the 3^rd^ day of the experiments; (c) the 5^th^ day of the experiments; (d) the 7^th^ day of the experiments; (e) the 10^th^ day of the experiments; (f) the 15^th^ day of the experiments.

Severe hyperemia and puffiness of the skin tissue were observed at the verge of the wound surface (Figures-1a-3a).

During treatment on the 3^rd^ and 5^th^ days of the experiment, a decrease in inflammation and puffiness at the edge of the wound was observed, and the first signs of healthy granulation tissue appeared in the form of separate focal areas. Eventually, it filled up the entire wound in all three experimental groups (Figures-1b and c-3b and c).

Subsequently, the transition of granulation tissue from bright red to pink characterized the proliferation and formation of the epithelium, which grew from the periphery to the center of the wound, covering the wound bed, which indicated the beginning of wound shrinkage. The growth of granulation tissue led to decreased wound depth, and epithelial cell growth led to a decreased wound area.

**Figure-4:**
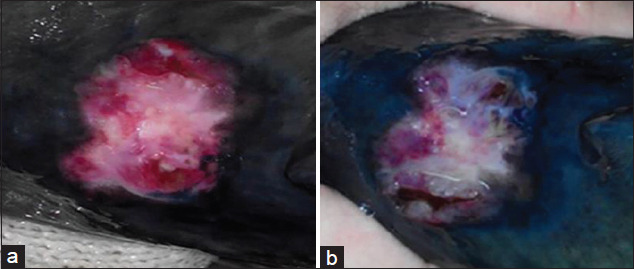
A wound in the control group (without treatment): (a) At the beginning of the experiment; (b) at the end of the experiment.

In the control group, inflammation and puffiness at the edge of the wound surface did not resolve, and excessive mucoid discharge was noted. In most cases, signs of necrosis and sloughing developed, and ulcers became larger in several individuals. There was no reduction in size or contraction of the wound (Figures-4a and b). Fish died in the case of transition to a generalized form (Table-1).

**Table-1:** Fish survival during the experiment.

Groups	The period of the experiment (days)						Survival rate, %

	3^rd^ day	5^th^ day	7^th^ day	10^th^ day	15^th^ day	20^th^ day	

	Fish mortality						
First group (n=15)	-	-	-	-	-	-	100
Second group (n=15)	1	-	-	-	-	-	93
Third group (n=15)	-	-	-	-	-	-	100
Control group (n=15)	4	2	1	1	-	-	46

Table-1 shows that eight fish died in the control group, and the survival rate was 46%. All fish survived (100% survival) in the first and third experimental groups. One fish died (93% survival) in the second group.

During the experiment, an improvement in the general condition of the fish and the clinical signs of disease disappeared in all experimental groups. The fish willingly ate the feed. In the control group, worsening illness, feed refusal, and an increase in the external symptoms of the disease were observed.

The wound process transition from the formation of granulation tissue to epithelization and regeneration was observed in all three experimental groups, indicating the effectiveness of the herbal infusions. However, the treatment effect on the change in wound surface area was significantly more pronounced in *C. majus* and *H. perforatum* groups, respectively (Table-2).

**Table-2:** The dynamics of wound healing in fish.

Groups	The average area of the wound (mm)							Wound healing, %

	Start	3^rd^ day	5^th^ day	7^th^ day	10^th^ day	15^th^ day	20^th^ day	
First group (n=15)	28.72±6.22	27.20±5.84**	23.91±5.09**	16.71±4.17	9.55±3.09	4.79±1.71	1.89±0.73	93.4
Second group (n=15)	20.39±4.37	17.23±4.24	16.24±4.03	15.77±4.00	15.33±3.93	14.86±3.93	14.39±3.91	29.5
Third group (n=15)	25.00±5.80	20.15±5.26	15.48±4.45	11.12±3.38	7.43±2.50	3.80±1.47	1.67±0.65	93.3
Control group (n=15)	19.03±4.31	11.47±3.15	8.60±2.93	8.24±3.20	7.57±3.24	7.84±3.33	8.19±3.58	0

*p<0.05;

** p<0.01; ***p<0.001 in comparison with the control

In the study of wound healing dynamics on the surface of the body of sick fish, a change in wound surface area was observed in the first and third experimental groups compared to the second experimental and control groups. Starting from the 7^th^ day after the beginning of the experiments, the groups treated with celandine and St. John’s wort showed a substantial reduction in wound size. The decrease in the wound surface area was 41.8% in Group 1 and 55.2% in Group 3. Subsequently and throughout the observation period (up to 20 days), the wound surface area in these groups continued to decrease (Figures-1d-f and 2d-f).

The maximum values by the 15^th^ and 20^th^ days of the experiments were found in Groups 1 and 3 (4.79±1.71 mm^2^ [83.3%] and 1.89±0.73 mm^2^ [93.4%]; and 3.80±1.47 mm^2^ [84.8%] and 1.67±0.65 mm^2^ [93.3%], respectively).

It should be noted that at all times during the experiments, although there was no pronounced effect on wound size in Group 2, the effect of the medicinal infusion on the healing process was similar to wound healing in the groups treated with celandine and St. John’s wort: A marked decrease in inflammation, the recession of puffiness, intensive formation of granulation tissue, and epithelization of wounds with slight scar formation toward the center of the wound, which was almost completed by the end of the experiments (Figures-3d-f). The wounds were closed with an epithelium with a relatively small reduction in area.

Therefore, bur beggar-ticks have a vulnerary effect, as do celandine and St. John’s wort, which contribute to the healing process.

**Figure-5:**
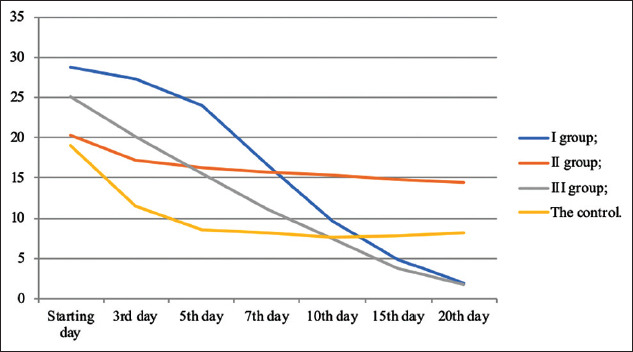
The dynamics of wound size reduction, mm^2^.

Figure-5 shows that the reduction in wound size in the first and third groups was significant compared with the second and control groups, which indicated intensive healing.

A visual assessment of wounds showed a decrease in inflammatory processes, granulation tissue growth, epithelial growth, tissue regeneration, and remolding of the tissues in the first, second, and third groups. The site of the wound remained slightly darker than the skin. Wound size decreased with the formation of granulation tissue.

The reduction of the wound in the first and third groups began by the 3^rd^ and 5^th^ day with granulation tissue formation and the formation and migration of epithelial cells from the edges to the center of the wound. A slight reduction in the wound size was observed in the second group with a pronounced vulnerary process.

Compared to the control group, the vulnerary process was more pronounced in the groups treated with *C. majus*, *H. perforatum*, and *B. tripartita* L. The wound size reduction was more pronounced in the first and third groups than in the second and control groups.

The results demonstrate that *C. majus* and *H. perforatum* infusions reduced the surface area of wounds better than bur beggar-tick and control. *Bidens tripartite* infusion and *C. majus* and *H. perforatum* infusions had a healing effect on the wound but did not significantly affect wound size.

No cases of recurrent disease were registered during the observation period for fish from the experimental groups.

In the available literature, there is a significant amount of scientific research on the use of medicinal plants as immunostimulants that increase disease resistance in fish by enhancing their non-specific and specific immunity [15-21]. However, there are very few studies on the wound healing effect of herbal drugs on spontaneously emerging wound pathologies of bacterial etiology in fish under real-world conditions in an aquarium complex. There are separate studies on herbal drugs for artificially simulated wound pathologies in fish [31-33].

Our research indicates that the proposed types and forms of herbal preparations, characterized by bacteriostatic and bactericidal action, promote the activation of non-specific immune mechanisms and have a pronounced wound healing effect. Herbal preparations, unlike chemotherapeutic ones, are ecologically safe and do not adversely affect the quality and nutritional value of fish products. These findings will be useful for the development of environmentally friendly strategies for controlling fish disease in aquaculture.

## Conclusion

This study showed a positive vulnerary effect of *C. majus*, *H. perforatum*, and *B. tripartita*, consistent with the use of these plants in folk medicine and traditional medicine. The use of raw plant material as a therapeutic agent eliminated infection, had anti-inflammatory and vulnerary effects, stimulated the development of granulation tissue, and enhanced the regeneration process due to the content of biologically active components in medicinal plants. Each biologically active agent may have a specific function and affect one or more phases of the vulnerary process.

A comparative study of the vulnerary properties of *C. majus*, *H. perforatum*, and *Bidens tripartite* with bacterial pathologies of sturgeon showed that infusions of *C. majus* and *H. perforatum* were the most promising. *Bidens tripartite* infusion also had a healing effect on the wound, but no noticeable effect on reducing the wound size was observed; however, we recommend using this herbal decoction as an auxiliary substance in complex therapeutic and prophylactic measures.

Compared with traditional methods (antibiotics and other chemotherapy methods), botanical therapy allows treatment to be carried out more effectively and as quickly as standard therapeutics but without unwanted side effects. The practical use and the results of this study show the potential of using herbal infusions as effective, safe, and affordable means for therapeutic purposes in treating fish in aquaculture.

## Authors’ Contributions

FN: Acquisition of data, conducted experiments, analysis and interpretation of data, and drafted the manuscript. GA: Conception and design, supervised the research, and final approval. BS: Procedure for conducting experiments, revision, and editing the article. AS: Comparison of research results and revised the article critically. NG: Statistical analysis and interpretation of data, and drafted the manuscript. KM: Selection of literature, creating a reference list, analysis, and interpretation of data. All authors have read and approved the final manuscript.
